# Breast density effect on the sensitivity of digital screening mammography in a UK cohort

**DOI:** 10.1007/s00330-024-10951-w

**Published:** 2024-07-17

**Authors:** Nicholas R. Payne, Sarah E. Hickman, Richard Black, Andrew N. Priest, Sue Hudson, Fiona J. Gilbert

**Affiliations:** 1 Department of Radiology, University of Cambridge School of Clinical Medicine, Box 218, Level 5, Cambridge Biomedical Campus, Cambridge, CB2 0QQ UK; 2 Department of Radiology, Barts Health NHS Trust, The Royal London Hospital, 80 Newark Street, London, E1 2ES UK; 3 Department of Radiology, Addenbrookes Hospital, Cambridge University Hospitals NHS Foundation Trust, Cambridge, UK; 4 Peel and Schriek Consulting Limited, London, UK

**Keywords:** Mammographic breast density, Digital mammography, Early detection of cancer

## Abstract

**Objectives:**

To assess the performance of breast cancer screening by category of breast density and age in a UK screening cohort.

**Methods:**

Raw full-field digital mammography data from a single site in the UK, forming a consecutive 3-year cohort of women aged 50 to 70 years from 2016 to 2018, were obtained retrospectively. Breast density was assessed using Volpara software. Examinations were grouped by density category and age group (50–60 and 61–70 years) to analyse screening performance. Statistical analysis was performed to determine the association between density categories and age groups. Volumetric breast density was assessed as a binary classifier of interval cancers (ICs) to find an optimal density threshold.

**Results:**

Forty-nine thousand nine-hundred forty-eight screening examinations (409 screen-detected cancers (SDCs) and 205 ICs) were included in the analysis. Mammographic sensitivity, SDC/(SDC + IC), decreased with increasing breast density from 75.0% for density a (*p* = 0.839, comparisons made to category b), to 73.5%, 59.8% (*p* = 0.001), and 51.3% (*p* < 0.001) in categories b, c, and d, respectively. IC rates were highest in the densest categories with rates of 1.8 (*p* = 0.039), 3.2, 5.7 (*p* < 0.001), and 7.9 (*p* < 0.001) per thousand for categories a, b, c, and d, respectively. The recall rate increased with breast density, leading to more false positive recalls, especially in the younger age group. There was no significant difference between the optimal density threshold found, 6.85, and that Volpara defined as the b/c boundary, 7.5.

**Conclusions:**

The performance of screening is significantly reduced with increasing density with IC rates in the densest category four times higher than in women with fatty breasts. False positives are a particular issue for the younger subgroup without prior examinations.

**Clinical relevance statement:**

In women attending screening there is significant underdiagnosis of breast cancer in those with dense breasts, most marked in the highest density category but still three times higher than in women with fatty breasts in the second highest category.

**Key Points:**

- *Breast density can mask cancers leading to underdiagnosis on mammography.*
- *Interval cancer rate increased with breast density categories ‘a’ to ‘d’; 1.8 to 7.9 per thousand.*
- *Recall rates increased with increasing breast density, leading to more false positive recalls.*

## Introduction

Breast cancer is one of the most common forms of cancer and early-stage diagnosis leads to better survival [1]. Population screening programmes aim to detect cancer early and reduce mortality. The UK National Health Service Breast Screening Programme (NHSBSP) invites women aged 50 to 70 years every 3 years for full-field digital mammography (FFDM). The longer screening interval results in more cancers diagnosed after a normal screening episode compared to many countries with a 2-yearly frequency of screening.

Breast density is important as it can mask or hide small cancers lowering the sensitivity of mammography—these cancers are often regarded as “underdiagnosed” cancers. Extremely dense breast tissue confers a fourfold relative risk of developing breast cancer compared to the lowest-density tissue [2, 3]. Breast density can be measured by readers on a visual analogue scale (VAS), categorised on the American College of Radiologists (ACR) Breast Imaging Reporting and Data System (BI-RADS) Atlas Fifth Edition four-point scale [4] or it can be variously assessed by automated tools.

In this study, we aimed to objectively assess the impact of breast density on the sensitivity and specificity of screening mammography and determine the rate of interval cancers (ICs) in each category of breast density using an automated tool. A secondary aim was to determine the optimal threshold when using volumetric breast density (VBD) as a binary classifier, to yield the highest ability to discriminate IC cases within the full cohort and within different age bands, 50–60 and 61–70 years.

## Materials and methods

Data were obtained from a single site in the UK where a four-view mammographic imaging protocol is used with cranial-caudal (CC) and mediolateral oblique (MLO) views of each breast. Double-reading with arbitration is undertaken within NHS Breast Screening Programme (NHSBSP) guidelines [5]. Ethical approval (Health Research Authority (HRA) Research Ethics Committee 20/LO/0104, HRA Confidentially Advisory Group (CAG) 20/CAG/0009, and Public Health England Research Advisory Committee BSPRAC_090) was obtained to retrospectively collect data from women who took part in breast screening during the years 2011 to 2020 without obtaining explicit consent from the individuals but with an ability for them to opt-out of their data being used.

The inclusion criteria for this study were mammograms collected as part of breast cancer screening at the local site between 2016 and 2018. Examinations were excluded from analysis if the images could not be obtained from the local picture archiving and communication system (PACS) or if raw DICOM data were not available; if the woman was outside of the 50–70 years age range of routine screening; if the woman had a history of breast cancer with a mastectomy or was undergoing annual screening; or if it was not the first examination of the same woman within the period. Examinations were also excluded if the automated density software was not able to score them and breast cancer cases were excluded if they were not the primary cancer.

### Data collection

Consecutive mammographic data from a 3-year cohort from 1st January 2016 to 31st December 2018 was selected to reflect the triennial screening cycle, when raw mammographic data was routinely stored at the site, and provide follow-up to identify all ICs. The FFDM images were acquired on Philips (Philips Healthcare) L30 scanners (~98%) and a small number on GE (GE HealthCare Technologies Inc.) machines. National Breast Screening Service (NBSS) records were used to query systems for imaging and clinical data. Imaging data and related metadata were obtained from the local PACS. The data was pseudonymised and stored in a research database.

All cancer cases were confirmed from histopathology reports. For normal (non-cancer) cases, ground truth was taken as no cancer diagnosis recorded in the National Cancer Registry before the next round of screening they attended or within 40 months if they did not re-attend. Data was collected up to April 2022 giving a minimum follow-up period of 40 months for all women. Ethnicity data, where available, was captured from a combination of NBSS and hospital records.

Screening exams are classified as either screen-detected cancer (SDC), for those diagnosed at screening, or IC if diagnosed between screening episodes and within 40 months of a normal screen. The case is classified as normal if there was no cancer diagnosis within the follow-up period. IC cases are identified by NBSS using a variety of methods, including through breast cancer data recorded by the National Cancer Registry, and then checked by individual screening centres. Histopathology (type, grade, and size) was also obtained from these sources where available. All ICs were symptomatic and none were detected by other screening methods.

### Breast density assessment

Automatic assessment of radiographic breast density was performed using Volpara Imaging Software’s density measure (v.3.2.0, Volpara Health Technologies Ltd) [6]. The software requires raw, uncompressed mammograms with DICOM headers containing exposure parameters for its physics-based model to compute the percentage VBD per image. The tool takes the maximum VBD at breast level and converts it to a Volpara Density Grade (VDG) for the case, which has been calibrated to correlate with BI-RADS 5th Edition breast density (a. almost entirely fat (VBD < 3.5%) b. scattered fibroglandular densities (3.5% ≤ VBD < 7.5%) c. heterogeneously dense, (7.5% ≤ VBD < 15.5%) and d. extremely dense (15.5% ≤ VBD)). This model has been validated in several previous studies [7–11].

### Exclusions

**Fig. 1:**
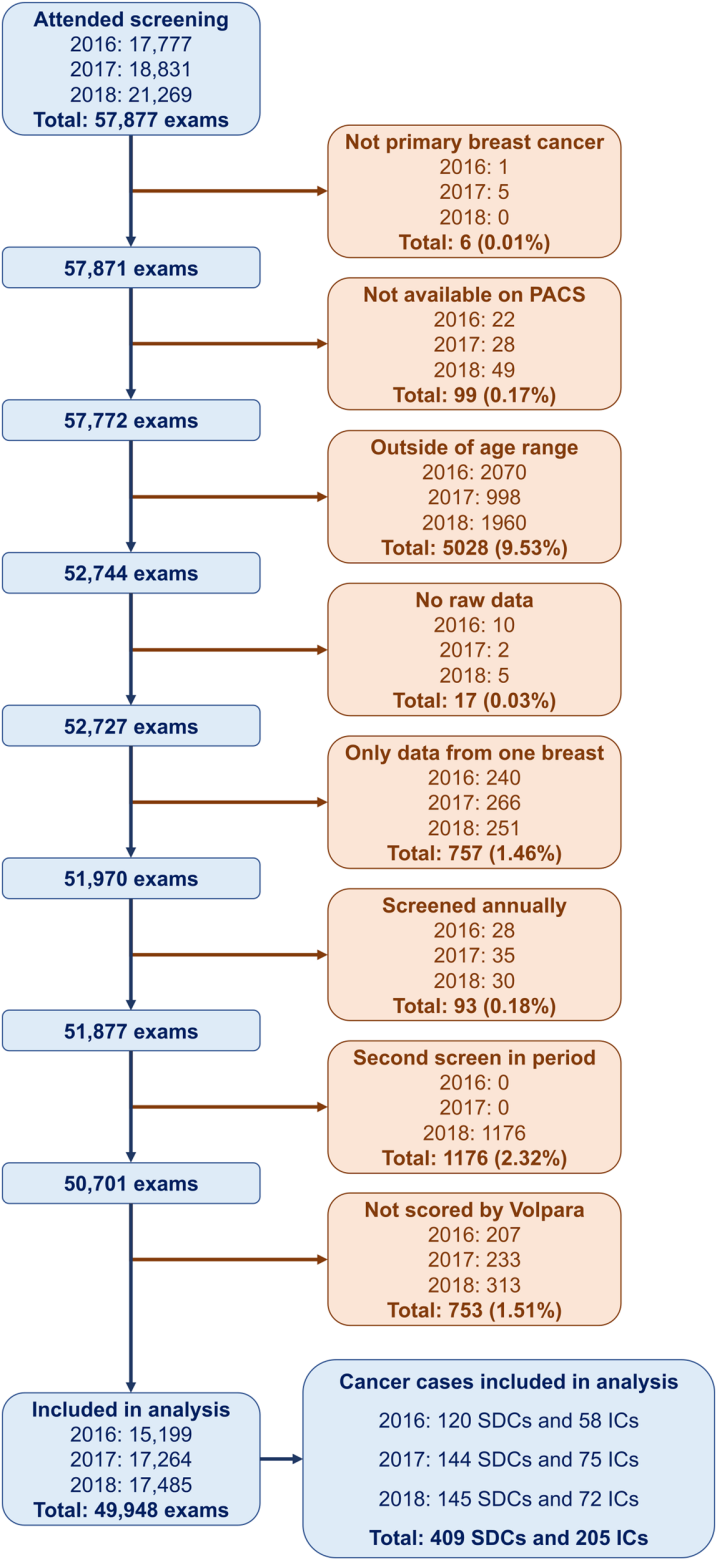
STARD (Standards for Reporting of Diagnostic Accuracy Studies) flow chart describing the exclusion of exams from the final analysis

Between 2016 and 2018, 57,877 women attended screening at the site. Six exams originally denoted as ICs were excluded as they were not primary breast cancers. There were 99 (0.17%) screening examinations not obtainable through the local PACS. A further 5028 (9.53%) were excluded as outside of the 50–70 age range. No raw data was available for 17 (0.03%) examinations. Women with a personal history of breast cancer with a mastectomy 757 (1.46%) or undergoing annual screening 93 (0.18%) were removed. Only the first screening episode of each woman within the cohort was used within the analysis, this led to the exclusion of 1176 (2.32%) of the remaining examinations as, in practice, the 3-year round length is variable and can be under 36 months. A total of 50,701 examinations were processed by the Volpara software of which 753 (1.51%) were not able to be scored—some exams received multiple errors and of those given; 79% were due to the presence of breast implants, 9% were parameter issues in data taken or missing from the DICOM header, 7% were mosaic/extended views (taking multiple images per view to fully cover the breast), and 5% were missing a view or side label. The final analysis was based on the remaining 49,948 examinations (Fig. 1).

### Statistical analysis

In the context of breast cancer screening, a true positive case is an SDC, a false positive is a recall with no cancer diagnosis before the next screen (or after 40 months of follow-up if not screened again), a false negative is an IC, and a true negative is a normal examination which was not recalled. For this study, we use the definition of the sensitivity of screening as the number of SDCs divided by the sum of screen-detected and ICs. The specificity of screening is the number of true negatives divided by the sum of true negatives and false positives. The positive predictive value (PPV) is given as the number of SDCs divided by the sum of SDCs and false positives.

Exams were grouped by Volpara Density Grade to measure the effect of breast density on screening performance and the distribution of density within the cohort. The cohort was then subdivided into two groups by age, those aged 60 and younger and those older than 60 years, to assess if either group was disproportionally affected by the impact of density. A two-tailed, two-sample *t*-test was used to determine the statistical significance between rates of SDC and IC of each density category when compared to that of the most populous/common density category. The same test was used to compare rates for the same density classification between the two age groups.

To investigate the impact of prevalent (baseline) round versus incident round on recall rates, the full cohort, the two age subgroups, and the four density subgroups were subdivided by round status. In each case, a two-tailed, two-sample *t*-test was used to determine the statistical significance between the recall rates of prevalent round examinations and incident round examinations.

VBD, volumetric density measured on a continuous scale (0 to 100%), was analysed as a binary classifier for identifying IC cases—investigating its potential as a metric to be used to determine which woman may benefit from enhanced screening (supplemental imaging or more frequent screening). Receiver operating characteristic (ROC) curves were computed with R (v. 4.3.0, R Foundation for Statistical Computing) with the pROC package [12] to find the area under the ROC curve (AUC) and optimal operating point/threshold—taken as the closest point to the top left of the chart with no case weighting. We calculated an overall threshold as well as separate thresholds for each age subgroup.

## Results

### Density distribution

A total of 49,948 women with screening examinations were included in the final analysis, comprising of 409 SDCs, 205 ICs, and 49,336 normals with a mean age of 59.0 years (±6.1 years). The overall density distribution was 17.3% a, 46.4% b, 26.6% c, and 9.7% d with women aged ≤ 60 and > 60 have the following proportions: 15.6% and 19.9% a; 43.1% and 51.5% b; 28.9% and 23.0% c; and 12.4% and 5.6% d, respectively.

### Sensitivity of screening

The 3 yearly sensitivity of mammography was highest in density a (75.0%), which was not found to be statistically significantly different from that of category b (*p* = 0.839). However, compared to category b, the sensitivity was worse in categories c and d which were found to be 59.8% (*p* = 0.001) and 51.3% (*p* < 0.001), respectively.

A full breakdown of screening performance by density is given in Table 1 and a further breakdown by density and age is given in Table 2. The lowest sensitivity of 48.1% was found for women aged 60 and under with category d density. Women with fatty breasts had significantly lower SDC rates (*p* = 0.002) than any other category with twice the rate for the older woman compared to those aged 60 and under (*p* = 0.009).

**Table 1:** Number of screening examinations, breast cancer cases, false positives, and true negatives for breast cancer screening at a single UK breast screening site 2016–2018 categorised by density

	Volpara Density Grade (Volpara 3.2.0)				
	a	b	c	d	Total
Screening exams (*N*) (%)	8658 (17.3%)	23,183 (46.4%)	13,274 (26.6%)	4833 (9.7%)	49,948 (100%)
Mean age (years) (mean ± SD)	60.0 (54.2–65.8)	59.7 (53.7–65.7)	58.2 (52.1–64.3)	56.2 (50.4–62.0)	59.0 (52.9–65.1)
SDC (*N*) (%)	48 (11.7%)	208 (50.9%)	113 (27.6%)	40 (9.8%)	409 (100%)
IC (*N*) (%)	16 (7.8%)	75 (36.6%)	76 (37.1%)	38 (18.5%)	205 (100%)
FP (*N*) (%)	207 (11.2%)	824 (44.5%)	609 (32.9%)	212 (11.4%)	1852 (100%)
TN (*N*) (%)	8387 (17.7%)	22,077 (46.5%)	12,476 (26.3%)	4544 (9.6%)	47,484 (100%)
Recalls (*N*) (%)	256 (11.2%)	1043 (45.7%)	728 (31.9%)	254 (11.1%)	2281 (100%)
SDC/1000 (95% CI) *p*-value	5.5 (4.0–7.1) *p* = 0.002	9.0 (7.8–10.2)	8.5 (6.9–10.1) *p* = 0.651	8.3 (5.7–10.8) *p* = 0.629	8.2 (7.4–9.0)
IC/1000 (95% CI) *p*-value	1.8 (0.9–2.8) *p* = 0.039	3.2 (2.5–4.0)	5.7 (4.4–7.0) *p* < 0.001	7.9 (5.4–10.4) *p* < 0.001	4.1 (3.5–4.7)
Recall rate (%) (95% CI) *p*-value	3.0 (2.6–3.3) *p* < 0.001	4.5 (4.2–4.8)	5.5 (5.1–5.9) *p* < 0.001	5.3 (4.6–5.9) *p* = 0.023	4.6 (4.4–4.7)
FP/1000 (95% CI) *p*-value	23.9 (20.7–27.1) *p* < 0.001	35.5 (33.2–37.9)	45.9 (42.3–49.4) *p* < 0.001	43.9 (38.1–49.6) *p* = 0.005	37.1 (35.4–38.7)
Sensitivity (95% CI) *p*-value	75.0% (64.4–85.6) *p* = 0.839	73.5% (68.4–78.6)	59.8% (52.8–66.8) *p* = 0.001	51.3% (40.2–62.4) *p* < 0.001	66.6% (62.9–70.3)
Specificity (95% CI) *p*-value	97.6% (97.3–97.9) *p* < 0.001	96.4% (96.2–96.6)	95.3% (95.0–95.7) *p* < 0.001	95.5% (95.0–96.1) *p* = 0.005	96.2% (96.1–96.4)
PPV (95% CI) *p*-value	18.8% (14.0–23.6) *p* = 0.634	20.2% (17.7–22.6)	15.7% (13.0–18.3) *p* = 0.016	15.9% (11.4–20.4) *p* = 0.123	18.1% (16.5–19.7)
NPV (95% CI) *p*-value	99.8% (99.7–99.9) *p* = 0.034	99.7% (99.6–99.7)	99.4% (99.3–99.5) *p* < 0.001	99.2% (98.9–99.4) *p* < 0.001	99.6% (99.5–99.6)

Screening performance metrics (95% CI) have also been calculated. All IC cases are taken as false negatives, including those recalled but not diagnosed (22 cases) Two-tail, two-sample *t*-tests were used for each performance metric listed in the first column of the lower half of the table (starting from SDC/1000) with *p*-values given—comparisons are made between each density category and the most populous category, b

*FP* false positive, *TN* true negative, *PPV* positive predictive value, *NPV* negative predictive value

**Table 2:** Number of screening examinations, breast cancer cases, false positives, and true negatives for breast cancer screening at a single UK breast screening site 2016–2018 categorised by VDG and age

	Volpara Density Grade (Volpara 3.2.0)									
	a		b		c		d		Total	
Age	≤ 60	> 60	≤ 60	> 60	≤ 60	> 60	≤ 60	> 60	≤ 60	> 60
Screening exams (*N*) (%)	4689 (9.4%)	3969 (7.9%)	12,931 (25.9%)	10,252 (20.5%)	8684 (17.4%)	4590 (9.2%)	3725 (7.5%)	1108 (2.2%)	30,029 (60.1%)	19,919 (39.9%)
SDC (*N*) (%)	17 (4.2%)	31 (7.6%)	103 (25.2%)	105 (25.7%)	57 (13.9%)	56 (13.7%)	26 (6.4%)	14 (3.4%)	203 (49.6%)	206 (50.4%)
IC (*N*) (%)	9 (4.4%)	7 (3.4%)	38 (18.5%)	37 (18.0%)	53 (25.9%)	23 (11.2%)	28 (13.7%)	10 (4.9%)	128 (62.4%)	77 (37.6%)
FP (*N*) (%)	128 (6.9%)	79 (4.3%)	517 (27.9%)	307 (16.6%)	451 (24.4%)	158 (8.5%)	181 (9.8%)	31 (1.7%)	1277 (69.0%)	575 (31.0%)
TN (*N*) (%)	4535 (9.6%)	3852 (8.1%)	12,273 (25.8%)	9804 (20.6%)	8123 (17.1%)	4353 (9.2%)	3491 (7.4%)	1053 (2.2%)	28,422 (59.9%)	19,062 (40.1%)
Recalls (*N*) (%)	145 (6.4%)	111 (4.9%)	625 (27.4%)	418 (18.3%)	512 (22.4%)	216 (9.5%)	209 (9.2%)	45 (2.0%)	1491 (65.4%)	790 (34.6%)
SDC/1000 (95% CI) *p*-value	3.6 (1.9–5.3)	7.8 (5.1–10.5) *p* = 0.009	8.0 (6.4–9.5)	10.2 (8.3–12.2) *p* = 0.068	6.6 (4.9–8.3)	12.2 (9.0–15.4) *p* < 0.001	7.0 (4.3–9.7)	12.6 (6.1–19.2) *p* = 0.068	6.8 (5.8–7.7)	10.3 (8.9–11.7) *p* < 0.001
IC/1000 (95% CI) *p*-value	1.9 (0.7–3.2)	1.8 (0.5–3.1) *p* = 0.867	2.9 (2.0–3.9)	3.6 (2.4–4.8) *p* = 0.372	6.1 (4.5–7.7)	5.0 (3.0–7.1) *p* = 0.428	7.5 (4.7–10.3)	9.0 (3.5–14.6) *p* = 0.618	4.3 (3.5–5.0)	3.9 (3.0–4.7) *p* = 0.497
Recalls (%) (95% CI) *p*-value	3.1 (2.6–3.6)	2.8 (2.3–3.3) *p* = 0.418	4.8 (4.5–5.2)	4.1 (3.7–4.5) *p* = 0.006	5.9 (5.4–6.4)	4.7 (4.1–5.3) *p* = 0.004	5.6 (4.9–6.3)	4.1 (2.9–5.2) *p* = 0.043	5.0 (4.7–5.2)	4.0 (3.7–4.2) *p* < 0.001
FP/1000 (95% CI) *p*-value	27.3 (22.6–32.0)	19.9 (15.6–24.3) *p* = 0.025	40.0 (36.6–43.4)	29.9 (26.6–33.2) *p* < 0.001	51.9 (47.3–56.6)	34.4 (35.4–46.9) *p* < 0.001	48.6 (41.7–55.5)	28.0 (18.3–37.7) *p* = 0.003	42.5 (40.2–44.8)	28.9 (26.5–31.2) *p* < 0.001
Sensitivity (95% CI) *p*-value	65.4% (47.1–83.7)	81.6% (69.3–93.9) *p* = 0.146	73.1% (65.7–80.4)	73.9% (66.7–81.2) *p* = 0.788	51.8% (42.5–61.2)	70.8% (60.9–80.9) *p* = 0.008	48.1% (34.8–61.5)	58.3% (38.6–78.1) *p* = 0.457	61.3% (56.1–66.6)	72.8% (67.6–78.0) *p* = 0.003
Specificity (95% CI) *p*-value	97.3% (96.8–97.7)	98.0% (97.6–98.4) *p* = 0.027	96.0% (95.6–96.3)	97.0% (96.6–97.3) *p* < 0.001	94.7% (94.3–95.2)	96.5% (96.0–97.0) *p* < 0.001	95.1% (94.3–95.8)	97.1% (96.1–98.1) *p* = 0.004	95.7% (95.5–95.9)	97.1% (96.8–97.3) *p* < 0.001
PPV (95% CI) *p*-value	11.7% (6.4–17.0)	28.2% (19.8–36.6) *p* < 0.001	16.6% (13.7–19.5)	25.5% (21.3–29.7) *p* < 0.001	11.2% (8.5–14.0)	26.2% (20.3–32.1) *p* < 0.001	12.6% (8.0–17.1)	31.1% (17.6–44.6) *p* = 0.002	13.7% (12.0–15.5)	26.4% (23.3–29.5) *p* < 0.001
NPV (95% CI) *p*-value	99.8% (99.7–99.9)	99.8% (99.7–100) *p* = 0.861	99.7% (99.6–99.8)	99.6% (99.5–99.7) *p* = 0.392	99.4% (99.2–99.5)	99.5% (99.3–99.7) *p* = 0.399	99.2% (98.9–99.5)	99.1% (98.5–99.6) *p* = 0.648	99.6% (99.5–99.6)	99.6% (99.5–99.7) *p* = 0.452

Screening performance metrics (95% CI) have also been calculated. IC cases are taken as false negatives, including those recalled but not diagnosed (22 cases). Two-tail, two-sample *t-*tests were used for each performance metric listed in the first column of the lower half of the table (starting from SDC/1000) with *p*-values given—comparisons are made within density categories between age groups

### Interval cancer, screen-detected cancer, and recall rates

Of the 205 IC cases analysed, 15.1% (31/205) were diagnosed within 12 months of the negative screen, 34.6% (71/205) were diagnosed between 12–24 months, and 50.2% (103/205) were diagnosed after 24 months. The IC rate increased from 1.8/1000 in category a to 7.9/1000 in category d with a large number of ICs in category c (5.7/1000). The IC rates for categories c and d were found to be significantly different to that of category b (*p* < 0.001 in both cases). The highest rate of cancers ((SDC + IC)/1000) was found in d category density at 16.1/1000 with 14.2, 12.2, and 7.4 per thousand in density categories c, b, and a, respectively.

The recall rate was significantly higher in categories c and d (5.5% (*p* < 0.001) and 5.3% (*p* = 0.023), respectively) and lower in category a (3.0% (*p* < 0.001)) when compared to the most populous category, b (4.5%). When dichotomised by age the recall was consistently and significantly higher in the younger women in density categories b (*p* = 0.006), c (*p* = 0.004), and d (*p* = 0.043) as well as the overall (*p* < 0.001). The effect of prevalent (baseline) versus incident status is shown in Table 3 with a recall rate of 8.6% found for prevalent rounds and 4.2% for subsequent rounds (*p* < 0.001). Although this is mirrored in the two age subgroups, it should be noted that 8.3% of examinations in the analysis are from prevalent rounds and 92.5% of those are women in the younger cohort.

**Table 3:** Recall rates by screening round status (“P” = prevalent (baseline) or “I” = incident) for the full cohort (i), age subgroups (i), and density subgroups (ii)

(i)	Full cohort		≤ 60		> 60	
Round status	P	I	P	I	P	I
Exams *(N*)	4152	45,796	3840	26,189	312	19,607
Recalled (*N*)	355	1926	331	1160	24	766
Recall rate (%) (CI: 95%)	8.6 (7.7–9.4)	4.2 (4.0–4.4)	8.6 (7.7–9.5)	4.4 (4.2–4.7)	7.7 (4.7–10.7)	3.9 (3.6–4.2)
*p*-value	*p* < 0.001		*p* < 0.001		*p* < 0.001	

(ii)	Volpara Density Grade (Volpara 3.2.0)							
	a		b		c		d	
Round status	P	I	P	I	P	I	P	I
Exams *(N*)	543	8115	1618	21,565	1284	11,990	707	4126
Recalled *(N*)	38	218	157	886	112	616	48	206
Recall rate (%) (CI: 95%)	7.0 (4.9–9.1)	2.7 (2.3–3.0)	9.7 (8.3–11.4)	4.1 (3.8–4.4)	8.7 (7.2–10.3)	5.1 (4.7–5.5)	6.8 (4.9–8.6)	5.0 (4.3–5.7)
*p*-value	*p* < 0.001		*p* < 0.001		*p* < 0.001		*p* = 0.048	

Two-tail, two-sample *t*-tests were performed comparing the recall rate between the prevalent and incident groups, *p*-values are given. There was no statistically significant difference between age subgroups

### Volumetric breast density

**Fig. 2:**
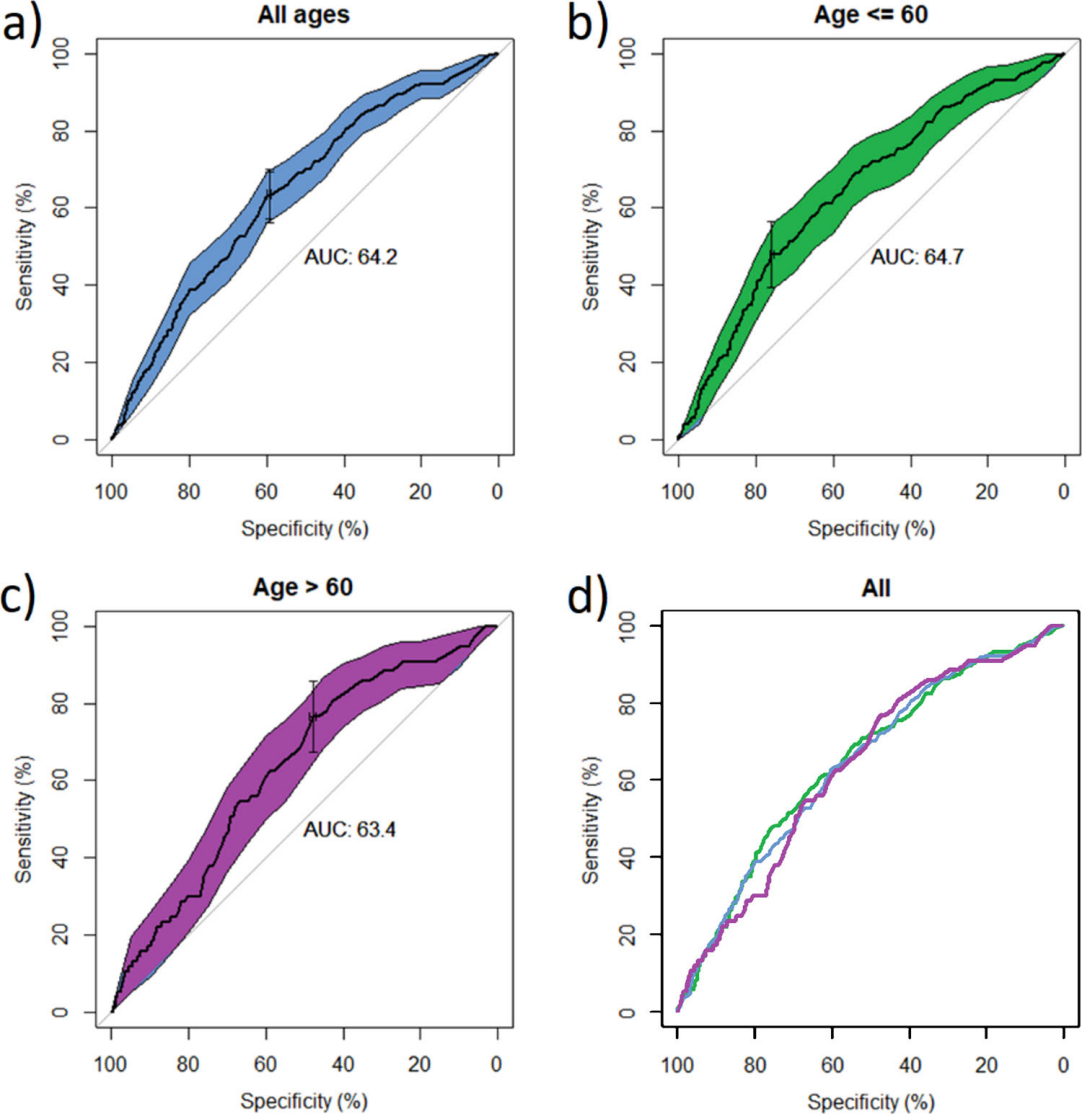
ROC curves for the performance of VBD as a binary classifier for IC in (**a**) the full cohort, (**b**) the subgroup aged 60 years and under, and (**c**) the subgroup aged over 60 years. 95% confidence intervals (coloured area) and optimal threshold are shown in each case. **d** Shows the three ROC curves together; the full cohort in blue, the subgroup aged 60 and under in green, and the subgroup aged over 60 in purple

When using VBD as a binary classifier to identify IC cases, the AUC for the full cohort (Fig. 2a) was found to be 64.2 (CI: 60.5–67.9) with an optimal threshold of 6.85 (CI: 4.65–9.85) corresponding to a sensitivity and specificity of 63.6% and 59.4%, respectively. This threshold defines 40.7% (20331/49948) as dense. For the younger subgroup alone (Fig. 2b) the AUC was 64.7 (CI: 60.0–69.4) with an optimal threshold of 8.15 (CI: 6.25–10.35), defining 35.1% (10538/30029) as dense. For the older subgroup alone (Fig. 2c) the AUC was 63.4 (CI: 57.6–69.3) with an optimal threshold of 5.15 (4.45–7.15), defining 52.2% (10397/19919) as dense. Using the optimal thresholds of the two subgroups gives a sensitivity and specificity of 65.0% and 58.2%, respectively—with a combined total of 41.9% (20,935/49,948) defined as dense. Using the thresholds of corresponding to VDG c and d, the sensitivities and specificity for each were found to be 55.8% and 63.8% (c and d) and 18.4% and 90.3% (d only), respectively.

### Additional cohort data

Ethnicity information was recorded in only 59.1% of the cohort of which 95.3% were listed as “White—British”, “White—Irish”, or “White—Any other White background”. The next highest ethnicities represented were “Other ethnic groups—Chinese” and “Asian or Asian British—Any other Asian background” each of which was 0.5% of the cohort. This is tabulated in the supplemental material.

**Fig. 3:**
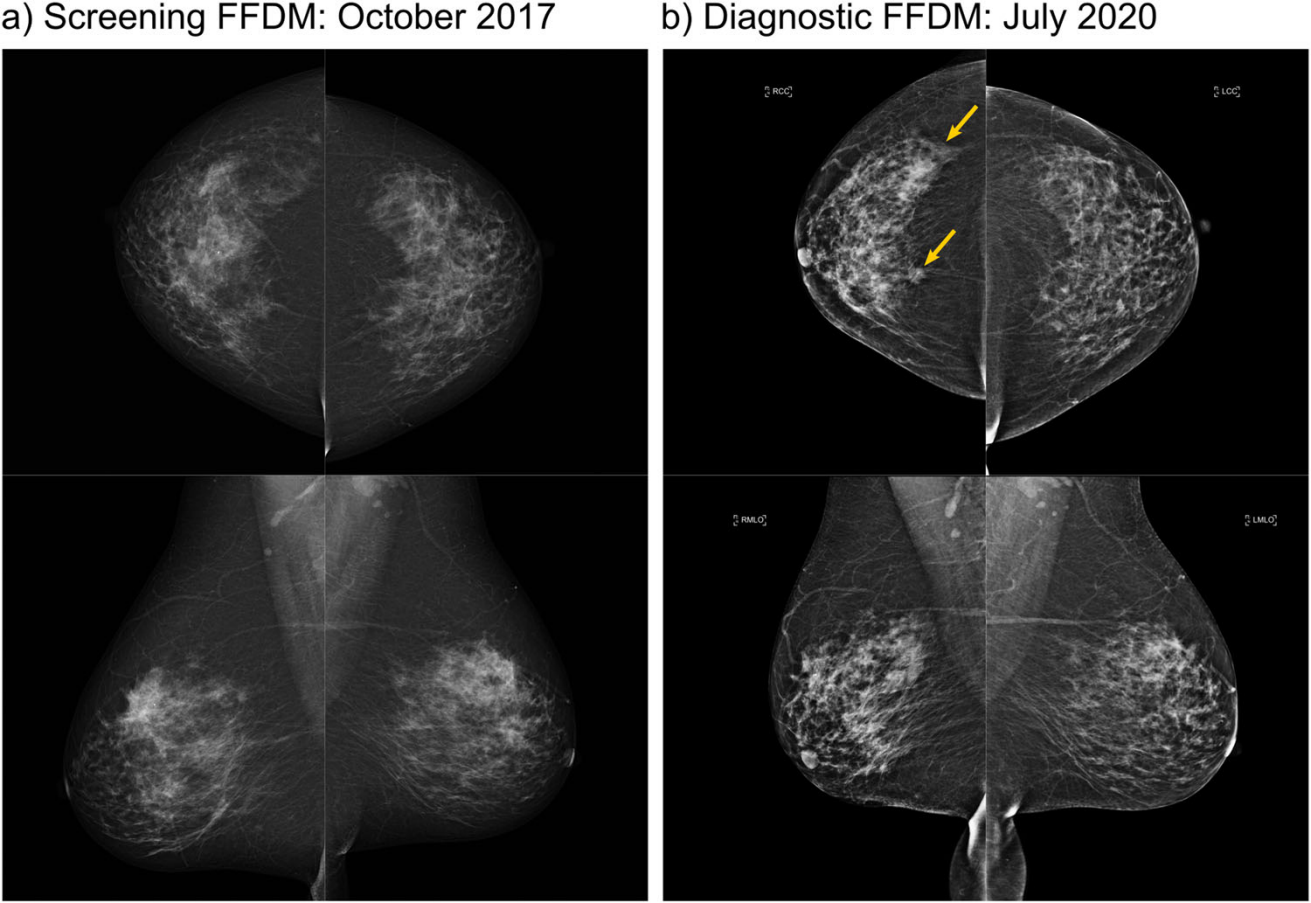
An example of an interval cancer case showing (**a**) the screening FFDM taken in October 2017 and (**b**) the diagnostic FFDM taken in July 2020. The patient underwent a right mastectomy and axillary clearance for a 120 mm Grade 2 invasive lobular carcinoma

For the 205 IC cases within the cohort, the number, median size, and interquartile range (IQR) of size are given by grade, interval, and VDG. This data can be found in the supplemental material. An example case of IC is shown in Fig. 3.

## Discussion

This study demonstrates that mammographic sensitivity drops dramatically with increasing breast density and in the 9.7% of the population with extremely dense breasts the sensitivity is only 51.3%. The results are similar to the Dutch biennial breast screening programme which reported a sensitivity of 61.0% in the 8.0% of women with extremely dense breasts [13]. The UK data appears to reflect the longer screening interval, with higher rates of both screen-detected and ICs as well as lower sensitives of screening. These similar findings between countries, with biennial and triennial breast screening programmes, provide evidence that increasing breast density is associated with a decrease in the screening performance of FFDM.

Recall rate increased with increasing breast density and was very similar at 5.5% and 5.3% in the densest two categories. False positive recalls are harmful for women causing short-term distress and costly to the screening programme. Digital breast tomosynthesis (DBT) has been shown to reduce false positive recall rates in women with BI-RADS c and d [14] as this reduces confusing overlapping shadows.

There are a number of ways to measure breast density. Reader assessment is subject to bias and inter-reader variability is marked particularly when the density category is borderline [15–17]. Automated tools are more self-consistent [18] but there is still concern about variability in performance and utility when comparing one with another [7]. Only a single-density tool, Volpara, was used in this study, but other studies have shown broad agreement when comparing automated tools [19, 20]. Calibration to particular populations is important [21] and Volpara is a well-validated [7–11], widely used tool with a number of publications reporting this density measure [22–24].

The increased number of ICs in the densest categories is of major concern. While many of these cancers have developed during the 3-year interval others will have been “underdiagnosed”, i.e., not seen at the time of screening. The European Society of Breast Imaging (EUSOBI) published new recommendations in 2022 calling for women undergoing screening to be informed of their breast density and for those with extremely dense breasts to be offered MRI every 2–4 years [25]. The Dutch DENSE trial found supplemental MRI for women with extremely dense breast tissue, as scored by Volpara software, halved the IC rate compared with the standard of care [26]. The American and German trials of Abbreviated MRI compared to DBT in dense breasts showed a sensitivity of 95.7% and 39.1%, respectively, but with poor specificity for MRI [27]. In the UK, the BRAID trial is recruiting women from screening with BI-RADS density c or d and randomising them to receive standard-of-care or supplemental imaging with either MRI, automated breast ultrasound, or contrast-enhanced mammography [28]. Full protocol MRI in the Dutch trial was only deemed cost-effective at 4-yearly intervals [29] and so a trial of contrast-enhanced mammography and abbreviated MRI is now planned.

If a form of supplemental imaging were to be offered to the highest category (d) then 9.7% of women would be eligible—which includes 18.6% of IC cases—whereas also including the second highest category (c) would lead to over one third (36.2%) of the screening population being invited for supplemental imaging, including over half (54.4%) of ICs. However, not all of these ICs have been overlooked and not all would be detected using a supplemental technique. Such a high level of eligibility may make arguments for supplemental imaging challenging on health and economic grounds. It may be possible to instead define the proportion of women for whom supplemental imaging could be affordable and set a density threshold on that basis.

More recently AI tools have been developed which assess breast texture as well as density and these have been used to predict who might develop breast cancer within the next 2 to 5 years. The Mirai tool, for example, has been found to have an AUC slightly higher than traditional risk prediction methods such as Tyrer-Cuzick [30]. The Swedish Karma tool has been tuned for 2-year risk and identified those women at high risk of being diagnosed with breast cancer with an AUC of 0.73 for its image-based model [31]. This tool is being tested in the ScreenTrust MRI [32] prospective intervention study.

Where assessment of breast density and textural analysis may help to identify women who would benefit from supplemental imaging to overcome masking due to breast composition, risk prediction could identify those who would benefit from a higher frequency of screening. Indeed, with half of the IC cases being diagnosed over 24 months after their negative screening outcome in the cohort undergoing triennial screening, a change to a biennial programme would reduce the number of ICs markedly. Furthermore, a Canadian study [33] showed biennial programs which additionally offered annual screening to women with dense breasts had an improved annualised IC rate (0.89/1000) compared to those that did not (1.45/1000).

## Limitations

This study was conducted at a single site in the UK with only one density tool. However, the similarities between tools [20, 34] are such that a single, well-evaluated tool can be used to demonstrate that mammography underperforms with increased breast density. The software tested requires the raw mammographic data although developments are in hand to use it for presentation images. Raw data, which undergoes vendor and software-specific post-processing to generate “for presentation” images, are not routinely stored by many institutions and therefore may not be available for density tools. However, using the tool prospectively is less problematic as a measurement can be made and raw data is not stored.

The definition of an IC within a triennial screening programme and the inclusion in the calculation of screening sensitivity is also a limitation as it is not possible to know if all ICs have been identified—some women may have moved abroad prior to diagnosis, for example—and, more importantly, it is likely many were not present at the time of the screening examination. Alternatively, some women who received a normal outcome at the round of screening included in the study and were not an IC case went on to be diagnosed with SDC at their next round of screening. Data on these “next round” cancers was not presented or included in sensitivity or specificity calculations despite the possibility of some of those cancers being present on the screening round included in the study.

The FFDM images were predominantly obtained on machines from a single vendor. It may be that the performance will differ on other manufacturer’s machines although this has not been reported.

Ethnicity was collected on just over half the population but the predominantly white British population may have introduced racial bias to both density distribution and screening performance results.

## Conclusion

This study has shown in a consecutive screening cohort that mammographic sensitivity and specificity decrease with increasing breast density as measured by an automated tool. The IC rate of 7.9/1000 indicates that a significant proportion of cases are underdiagnosed and consideration should be given to offer supplemental imaging in category d density. The 3-yearly round length exacerbates the problem.

## Supplementary information

Supplementary Material

## Abbreviations

AUC: Area under the receiver operating characteristic curve
BI-RADS: Breast Imaging Reporting and Data System
FFDM: Full-field digital mammography
IC: Interval cancer
MRI: Magnetic resonance imaging
NBSS: National Breast Screening Service
NHSBSP: National Health Service Breast Screening Programme
PACS: Picture Archiving and Communication System
ROC: Receiver operating characteristic
SDC: Screen-detected cancer
VBD: Volumetric breast density
VDG: Volpara density grade
