# Supplementary material for: Breast density effect on the sensitivity of digital screening mammography in a UK cohort
Source: Eur Radiol. 2024 Jul 17;35(1):177–87. doi: 10.1007/s00330-024-10951-w (PMC11631811; doi:10.1007/s00330-024-10951-w)
Supplement: Supplementary file 1 — Supplementary Material [file 330_2024_10951_MOESM1_ESM.pdf]

Supplemental material

Interval cancer data

The interval between screen and diagnosis was categorised as "Year 1" for an interval cancer diagnosed within 12 months of the screen, "Year 2" for those in months 13 to 24, or "Year 3" for those in months 25-40. Histopathological size was used where available (n=154), with the exception of those who received neoadjuvant chemotherapy (n=36), otherwise size on mammography (n=32) or ultrasound (n=16) were used in that order. No size data was available in three cases. 178 cancers were noted as invasive, 13 were non-invasive, and for 14 it was not known.

Grade		Interval between screen and diagnosis			
		Year 1	Year 2	Year 3	Total
1	N	4	6	11	21
	Median size	14 mm	10 mm	10 mm	12 mm
	IQR	[11 – 14]	[5 – 12]	[9 – 19]	[8 – 15]
2	N	12	26	45	83
	Median size	20 mm	17 mm	23 mm	22 mm
	IQR	[10 – 27]	[15 – 45]	[17 – 35]	[15 – 35]
3	N	11	28	39	78
	Median size	24 mm	20 mm	27 mm	23 mm
	IQR	[19 – 44]	[15 – 28]	[20 – 35]	[18 -32]
No grade data	N	4	11	8	23
	Median size	27 mm	14 mm	18 mm	17 mm
	IQR	[25 – 35]	[9 – 17]	[14 – 90]	[12 – 30]
Total	N	31	71	103	205
	Median size	22 mm	17 mm	23 mm	21 mm
	IQR	[13 – 31]	[12 – 30]	[15 – 32]	[14 – 31]

Grade		Volpara Density Grade (VDG) a	b	c	d	Total
1	N<br>Median size<br>IQR	1<br>10 mm	10<br>12 mm<br>[9 – 17]	6<br>12 mm<br>[5 – 14]	4<br>9 mm<br>[8 – 20]	21<br>12 mm<br>[8 – 15]
2	N<br>Median size<br>IQR	5<br>15 mm<br>[12 – 75]	26<br>26 mm<br>[15 – 32]	35<br>21 mm<br>[15 – 32]	17<br>22 mm<br>[15 – 38]	83<br>22 mm<br>[15 – 35]
3	N<br>Median size<br>IQR	9<br>31 mm<br>[22 – 36]	29<br>22 mm<br>[16 – 29]	25<br>27 mm<br>[20 – 39]	15<br>21 mm<br>[10 – 28]	78<br>23 mm<br>[18 -32]
No grade data	N<br>Median size<br>IQR	1<br>No data	10<br>18 mm<br>[8 – 26]	10<br>23 mm<br>[17 – 107]	2<br>8 mm<br>[3 – 13]	23<br>17 mm<br>[12 – 30]
Total	N<br>Median size<br>IQR	16<br>23 mm<br>[14 – 38]	75<br>20 mm<br>[14 – 29]	76<br>21 mm<br>[15 – 36]	38<br>19 mm<br>[10 – 30]	205<br>21 mm<br>[14 – 31]

Interval		Volpara Density Grade (VDG) a	b	c	d	Total
Year 1	N<br>Median size<br>IQR	1<br>15 mm	11<br>24 mm<br>[13 – 30]	12<br>21 mm<br>[13 – 43]	7<br>22 mm<br>[18 – 25]	31<br>22 mm<br>[13 – 31]
Year 2	N<br>Median size<br>IQR	12<br>31 mm<br>[21 – 41]	23<br>15 mm<br>[11 – 21]	19<br>17 mm<br>[14 – 22]	17<br>17 mm<br>[12 – 32]	71<br>17 mm<br>[12 – 30]
Year 3	N<br>Median size<br>IQR	3<br>14 mm<br>[11 – 30]	41<br>23 mm<br>[17 – 31]	45<br>25 mm<br>[18 – 39]	14<br>19 mm<br>[7 – 31]	103<br>23 mm<br>[15 – 32]
Total	N<br>Median size<br>IQR	16<br>23 mm<br>[14 – 38]	75<br>20 mm<br>[14 – 29]	76<br>21 mm<br>[15 – 36]	38<br>19 mm<br>[10 – 30]	205<br>21 mm<br>[14 – 31]

Ethnicity data

**Ethnicity**		**In cohort N**	**(%)**
A	White – British	26590	(53.2%)
B	White – Irish	208	(0.4%)
C	White – Any other White background	1340	(2.7%)
D	Mixed – White and Black Caribbean	18	(0.0%)
E	Mixed – White and Black African	13	(0.0%)
F	Mixed – White and Asian	43	(0.1%)
G	Mixed – Any other Mixed background	77	(0.2%)

H	Asian or Asian British – Indian	207 (0.4%)
J	Asian or Asian British – Pakistani	53 (0.1%)
K	Asian or Asian British – Bangladeshi	37 (0.1%)
L	Asian or Asian British – Any other Asian background	247 (0.5%)
M	Black or Black British - Caribbean	87 (0.2%)
N	Black or Black British - African	110 (0.2%)
P	Black or Black British – Any other Black background	37 (0.1%)
R	Other ethnic groups – Chinese	273 (0.5%)
S	Other ethnic groups – Any other group	189 (0.4%)
Z	Not stated	3256 (6.5%)
	Not available	17163 (34.4%)
	**Total**	**49948 (100%)**
